# Theoretical investigation on switchable second-order nonlinear optical (NLO) properties of novel cyclopentadienylcobalt linear [4]phenylene complexes

**DOI:** 10.1007/s00894-012-1681-z

**Published:** 2013-01-11

**Authors:** Wen-Yong Wang, Xiao-Feng Du, Na-Na Ma, Shi-Ling Sun, Yong-Qing Qiu

**Affiliations:** Institute of Functional Material Chemistry, Faculty of Chemistry, Northeast Normal University, Changchun, 130024 People’s Republic of China

**Keywords:** CpCo linear [4]phenylene complex, DFT, Frequency dependent, NLO property

## Abstract

As a kind of novel organometallic complexes, the cyclopentadienylcobalt (CpCo) linear [4]phenylene complexes (4 = number of benzene rings) display efficient switchable nonlinear optical (NLO) response when CpCo reversibly migrates along the linear [4]phenylene triggered by heating or lighting. In this paper, the second-order NLO properties for CpCo linear [4]phenylene complexes were calculated by using the density functional theory (DFT) methods with four functionals. All of the functionals yield the same order of *β*_*tot*_ values: 1<2<4<3. The effect of solvent on second-order NLO properties has been studied using polarized continuum model (PCM) in the tetrahydrofuran (THF) solution. The solvent leads to a slight enhancement of the NLO responses for the studied complexes relevant to their NLO responses in vacuo. The electronic absorption spectra were investigated by the TDDFT methods. The TDDFT calculations indicate that the maximum absorption peaks of complexes 2–4 in the near-infrared spectrum area show the bathochromic shift together with a decreasing intensity compared to complex 1. We have also found that the cobalt (Co) atom acts as a donor in all the organometallic complexes and the d → π* and π → π* charge transfer (CT) transitions contribute to the enhancement of second-order NLO response. Furthermore, two experimentally existing complexes 1 and 3 are found to have a large difference in *β*_*tot*_ values. It is our expectation that this difference may stimulate the search for a new type of switchable NLO material based on CpCo linear [4]phenylene complexes.

**Figure:**
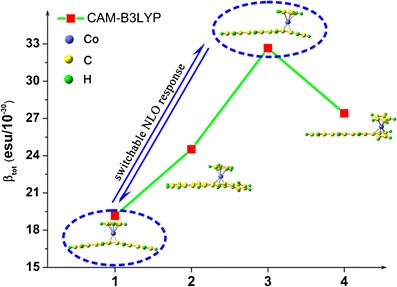
The second-order NLO properties of the cyclopentadienylcobalt (CpCo) linear [4]phenylene complexes were investigated by density functional theory (DFT) method, and complexes **1** and **3** display switchable NLO responses.

The second-order NLO properties of the cyclopentadienylcobalt (CpCo) linear [4]phenylene complexes were investigated by density functional theory (DFT) method, and complexes **1** and **3** display switchable NLO responses.

## Introduction

Nonlinear optical (NLO) materials have been developed rapidly due to their potential utility in optical data storage, optical communication, optical computing, biological imaging, signal processing, and image reconstruction technologies [1–6]. Great efforts have been devoted to obtain highly efficient NLO materials. It is well-known that the molecular second-order NLO properties can be manipulated by modifying the donor and acceptor capacity and extending the π-conjugated bridge. Lots of work has been focused on push-pull molecules containing a donor and an acceptor connected via a π-conjugated bridge (D-π-A) [7–9]. Generally, highly efficient NLO materials are mostly consistent with obvious charge transfer (CT) transitions. Thus, in most cases, the D-π-A structure is designed to enhance the CT transitions.

NLO activity can be found in organic compounds and inorganic crystals such as LiNbO_3_, and also in organometallic complexes [10, 11]. Organic materials have low-energy transitions in the UV–vis region which enhanced the NLO efficiency, but result in a tradeoff between nonlinear efficiency and optical transparency and they may have low thermal stability [12]. Inorganic crystals have several drawbacks: high quality single crystals are difficult to grow, are expensive, and are not easy to incorporate into electronic devices [11]. However, organometallic complexes offer greater scope for creation of multifunctional NLO materials by virtue of their greater design flexibility, low energy, and intense electronic transitions [12–14]. Since the 1980s, the investigations of the NLO properties for organometallic complexes have been especially arresting [15]. Those research works find that organometallic complexes can possess large NLO responses due to their lower transition energies and more intense CT transitions. Further, since the metal acts variously as a donor or an acceptor in the organometallic complexes, CT transitions of the organometallic complexes can be involved in three types: metal-to-ligand charge transfer (MLCT), ligand-to-metal charge transfer (LMCT) and metal-to-metal/intervalence charge transfer (MM/IVCT) [16]. In most cases, organometallic complexes have the noncentrosymmetric structure which is a universal requirement for the large first hyperpolarizability (*β*). The *β* value is often taken as a reference to establish NLO behavior.

Organometallic complexes also have the ability to switch their NLO properties through redox, deprotonation, turn on-off the conjugation, tautomerization reaction and so on [17, 18]. To reach the switchable characteristics, organometallic complexes must have two forms whose physical properties (or the *β* values) are significantly different, and their thermal and chemical properties are both stable [19]. The design of organometallic complexes with high switchable NLO responses has motivated a lot of experimental works. Coe et al. used hyper-rayleigh scattering (HRS) and electronic stark effect (electroabsorption) spectroscopic measurements to probe the quadratic NLO effects of a range of ruthenium complexes [14, 20]. Reversible Ru^II/III^ complexes are potentially redox-switchable chromophores [10, 21, 22]. Theoretical studies have also been performed to rationalize the switchable NLO properties. Liu has forecasted the switchable NLO properties of tetrathiafulvalene (TTF) derivatives by FF method [17, 23]. Theoretical work has enough ability to interpret the NLO property, even to design complexes with markedly switchable NLO responses in advance of experimental investigation.

**Fig. 1:**
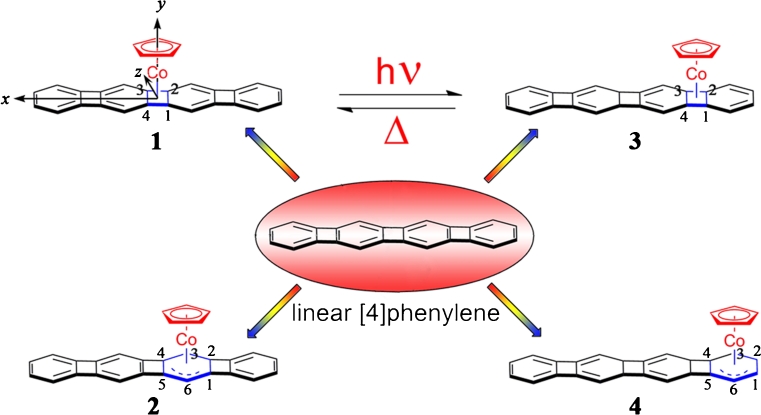
Chemical structures for complexes 1–4 and linear [4]phenylene

In this paper, we investigated the CpCo linear [4]phenylene complexes which display efficient switchable NLO properties. Vollhardt et al. first reported the CpCo linear [4]phenylene complexes, in which CpCo undergos thermally reversible photometallahaptotropism along the linear [4]phenylene between the inner and outer cyclobutadiene ring (1⇋3, see Fig. 1) [24]. To establish the path of CpCo migration, Vollhardt also computed the CpCo linear [4]phenylene complex 2 (whose CpCo is above the inner benzene ring). The CpCo linear [4]phenylene complexes may display switchable NLO properties, because the CpCo locates above the different cyclobutadiene and benzene rings. Nevertheless, a detailed understanding of the switchable NLO properties for the CpCo linear [4]phenylene complexes are still lacking. Hence, to better predict the switchable NLO responses of the CpCo linear [4]phenylene complexes, we designed complex 4 (whose CpCo is above the outer benzene ring) and studied the NLO properties of complexes 1, 2, 3 and 4. We hope this study may evoke the possibility to explore a new thriving area, i.e., CpCo linear [4]phenylene complexes for NLO application.

## Computational details

**Fig. 2:**
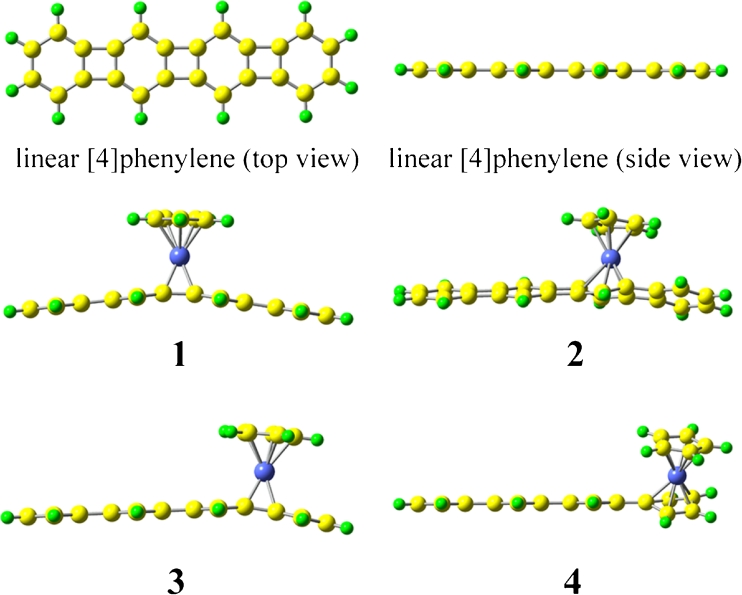
Optimized structures for complexes 1–4 (side view) and linear [4]phenylene

The geometrical structures of CpCo linear [4]phenylene complexes 1–4 and linear [4]phenylene (See Fig. 2) were obtained by density functional theory (DFT) method at B3LYP/6-31G(d) (LanL2DZ basis set for Co ion) level with real frequency. The B3LYP functional reproduces the geometries of molecules containing transitional metals very well [25, 26]. To obtain more accurate geometry, solvent effect has been taken into account in optimization and modeled using the polarized continuum model (PCM). We also optimized the geometrical structure of complex 1 in the tetrahydrofuran (THF) solution. The computed results (the Co-C bond lengths only increase ~0.002 Å) give very similar results to the vacuo calculations. This indicates that solvent effect on the geometrical structure is slight. Moreover, the calculation of natural bond orbital (NBO) analysis was performed at the B3LYP/6-31G(d) (LanL2DZ basis set for Co ion) level.

When a molecule is set in a uniform electric field *F*, its energy *E* can be described by the following formula:

1 $$ E(F)=E(0)-{\mu_i}{F_i}-\frac{1}{2}{\alpha_{ij }}{F_i}{F_j}-\frac{1}{6}{\beta_{ijk }}{F_i}{F_j}{F_k}-\frac{1}{24 }{\gamma_{ijkl }}{F_i}{F_j}{F_k}{F_l}-..... $$

Where *α*_*ij*_, *β*_*ijk*_ and *γ*_*ijkl*_ are the polarizability, the first hyperpolarizability and second hyperpolarizability tensors, respectively. The subscripts *i*, *j* and *k* label *x*, *y* and *z* components. It is clear that the values of *α*_*ij*_, *β*_*ijk*_ and *γ*_*ijkl*_ can be obtained by differentiating *E* with respect to *F*. In this work, the static first hyperpolarizability *β* was calculated by analytical third energy derivatives, which is more efficient and less expensive [27]. The total second-order polarizabilities (*β*_*tot*_) for the studied complexes are defined as:

2 $$ {\beta_{tot }}={{\left( {\beta_x^2+\beta_y^2+\beta_z^2} \right)}^{{{1 \left/ {2} \right.}}}}, $$

Where *β*_*i*_ is defined as:

3 $$ \begin{array}{*{20}c} {{\beta_i}=\left( {{\beta_{iii }}+{\beta_{ijj }}+{\beta_{ikk }}} \right)} \hfill & {i,j,k=x,y,z} \hfill \\ \end{array}. $$

To check the consistency of our calculation, the *β* value was calculated using Becke3-Lee-Yang-Parr (B3LYP) functional, long-range corrected (CAM-B3LYP) functional [28], half-and-half (BHandHLYP) functional [29] and new hybrid meta (M06-2X) functinal [30] at 6−31++G(d) (LanL2DZ basis set for Co ion) level. It is clearly seen that the four functionals display the same trend in *β* values. For clarity, we only take the CAM-B3LYP functional as an example to shed light on the changes in the first hyperpolarizabilities of the studied complexes. Furthermore, to understand the influence of the dispersion (frequency dependence) and the effect of the electron correlation on the NLO properties, the frequency-dependent *β* of CpCo linear [4]phenylene complexes were evaluated using couple-perturbed (CP) DFT method with CAM-B3LYP functional. In addition, the PCM model has been used to examine the solvent effect on the first static hyperpolarizabilities and frequency-dependent hyperpolarizabilities.

To further explain the second-order NLO behavior for the series of complexes, we employed time-dependent density functional theory (TDDFT) methods to descript their electronic spectra. TDDFT is one of the most accurate approaches for calculating the low-lying single excitations [31–33]. To choose suitable calculated methods, the electron absorption spectrum of complex 1 was simulated using TD-B3LYP, TD-CAM-B3LYP, TD-BHandHLYP, TD-M06-2X and TD-BP86 functionals with 6−31++G(d) (LanL2DZ basis set for Co ion) associated with PCM in THF solution. The maximum absorption peaks obtained by B3LYP, CAM-B3LYP, BHandHLYP, M06-2X and BP86 are 48 nm, 70 nm, 67 nm, 62 nm and 1 nm shorter than that of experimental data (385 nm), respectively. These results show that the absorption spectrum obtained by BP86 functional is in more reasonable agreement with the experimental data than other functionals. Also, to consider the solvent effects in absorption spectra, the absorption spectrum of complex 1 predicted by vacuo calculation has been performed on optimized geometry. The absorption spectrum of complex 1 predicted by vacuo calculation shows the blue shift (about 12 nm) compared to the PCM calculation. Therefore, the effect of the PCM on the absorption spectrum is strong and the absorption spectra for all studied complexes were calculated by using the TD-BP86 functional in THF solution.

All of the calculations in this work were carried out by using the Gaussian 09 W program package [34]. The molecular orbital compositions were analyzed using the AOMIX 6.52 [35, 36] program package. We divided linear [4]phenylene and CpCo into two fragments.

## Results and discussion

### Geometrical structure and natural bond orbital (NBO) analysis

It is well-known that the geometrical structure has a great effect on the properties of organometallic complexes. For our investigation herein, the origin of Cartesian coordinate system was located at the middle of the linear [4]phenylenes for all the studied complexes and the linear [4]phenylenes were placed in the *xz*-plane with longitudinal axis along the *x*-direction, while *y*-axis pointed to the Co ion and is orthogonal to cyclopentadienyl (see Fig. 1), and all the geometrical structures with real frequencies were obtained at the B3LYP/6-31G(d) (LanL2DZ basis set for Co ion) level. The results show that the energy of complex 1 is 16.7 kcal mol^−1^ more stable than that of complex 2 and is 6.6 kcal mol^−1^ more stable than that of complex 3, which is similar to the work of Vollhardt et al. (16.1 kcal mol^−1^ and 7.6 kcal mol^−1^, respectively). It is suggested that the optimized geometrical structures are reliable. From Fig. 2, it is clearly seen that the incorporation of CpCo has a significant influence on geometries of CpCo linear [4]phenylene complexes. In the optimized structures, we find that the structures of complexes 1 and 3 are bow-shaped with respect to linear [4]phenylene. Meanwhile, the structures of complexes 2 and 4 are twisted. This means that the geometrical structures of CpCo linear [4]phenylene complexes are different when CpCo is above the cyclopentadienyl and the benzene ring. In order to further understand the geometrical structures of CpCo linear [4]phenylene complexes, the selected bond lengths of Co-C between CpCo and linear [4]phenylene have been listed in Table 1. The bond length of 3 is the shortest among complexes 1–4, and the order of Co-C is: 3>1>2>4. This order demonstrates that various location of the CpCo remarkably affects the bond length.

**Table 1:** Selected bond lengths (Å) and NBO analysis of complexes 1–4

Complex	Bond length		Occupancy	Orbital coefficient
1	C1-Co	2.019	1.6656	0.6446sp^34.91^d^0.01^+0.7645sp^0.03^d^67.79^
	C2-Co	2.022	1.6646	0.6446sp^35.25^d^0.01^+0.7645sp^0.03^d^67.25^
2	C2-Co	2.090	1.7480	0.6720sp^12.79^d^0.00^+0.7405sp^0.03^d^73.55^
	C5-Co	2.113	1.7639	0.6849sp^12.50^d^0.00^+0.7286sp^0.03^d^58.72^
3	C3-Co	1.999	1.7008	0.6583sp^25.73^d^0.01^+0.7527sp^0.02^d^42.91^
	C4-Co	2.000	1.7009	0.6585sp^25.82^d^0.01^+0.7526sp^0.02^d^42.93^
4	C3-Co	2.124	1.7710	0.6620sp^17.63^d^0.01^+0.7495sp^0.06^d^64.30^

To interpret the bond character and the interaction between CpCo and linear [4]phenylene, natural bond orbital (NBO) analysis has been taken into account, and the results have also been shown in Table 1. It can be found that the bond of Co-C is formed by p orbital of C atom and d orbital of Co atom. The NBO charge of Co atoms for all complexes is about 0.5 a.u., and that of the linear [4]phenylene is about −0.2 a.u., as shown in Table 2. Therefore, we forecast that the CTs mainly occur between Co atom and linear [4]phenylene for all complexes.

**Table 2:** NBO charge of Co and linear [4]phenylene for complexes 1–4

Charge	1	2	3	4
Co	0.5172	0.5583	0.5289	0.5354
linear [4]phenylene	−0.2347	−0.2813	−0.2289	−0.2287

### Electronic structure and absorption spectrum

**Fig. 3:**
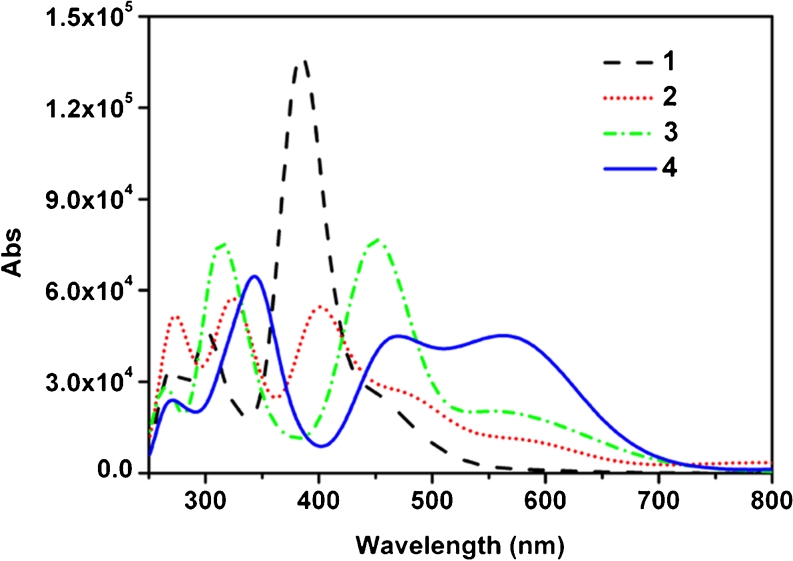
Calculated optical spectra for complexes 1–4

In order to obtain a more intuitive description of the band assignments of the electronic absorption spectra and the trends in the NLO behaviors of the studied complexes, TDDFT method has been performed on the excited states. The TDDFT calculated wavelengths (*λ*, nm), excited state transition energies (∆*E*_*gm*_, eV), oscillator strengths (*f*_*os*_), and major molecular orbital transitions of complexes 1–4 have been summarized in Table 3. The absorption spectra for complexes 1–4 have been plotted in Fig. 3. Complex 1 has a very strong maximum absorption peak at 385 nm. It is also noteworthy that the maximum absorption peaks of complexes 2–4 show the bathochromic shift together with a decreasing intensity compared to complex 1. Combined with Table 3, we can see the bathochromic shift of complex 2 is 11 nm, and a relatively larger bathochromic shift of complex 3 and complex 4 is 75 nm and 71 nm, respectively.

**Table 3:** Wavelengths (*λ*, nm), excited state transition energies (∆*E*_*ge*_, eV), oscillator strengths (*f*_*os*_), and major molecular orbital contributions of complexes 1–4 calculated using TDDFT method at the BP86/6-31++G(d) (LanL2DZ basis set for Co ion) level in the THF solution

Complex	State	λ	∆*E*_*ge*_	*f*_*os*_	Major contributions (%)
1	S_1_	696	1.78	0.0000	HOMO→LUMO(99 %)
	S_20_	385	3.22	1.7240	HOMO-5→LUMO(49 %), HOMO-2→LUMO+2(21 %)
2	S_1_	855	1.45	0.0135	HOMO→LUMO(82 %)
	S_19_	396	3.13	0.2910	HOMO→LUMO(18 %), HOMO-3→LUMO+3(16 %)
3	S_1_	771	1.61	0.0004	HOMO→LUMO(97 %)
	S_14_	460	2.70	0.6737	HOMO-2→LUMO+2(54 %), HOMO-5→LUMO(14 %)
4	S_1_	1001	1.24	0.0505	HOMO→LUMO (92 %)
	S_14_	456	2.72	0.3829	HOMO-1→LUMO+2(38 %), HOMO-1→LUMO+3(30 %)

**Fig. 4:**
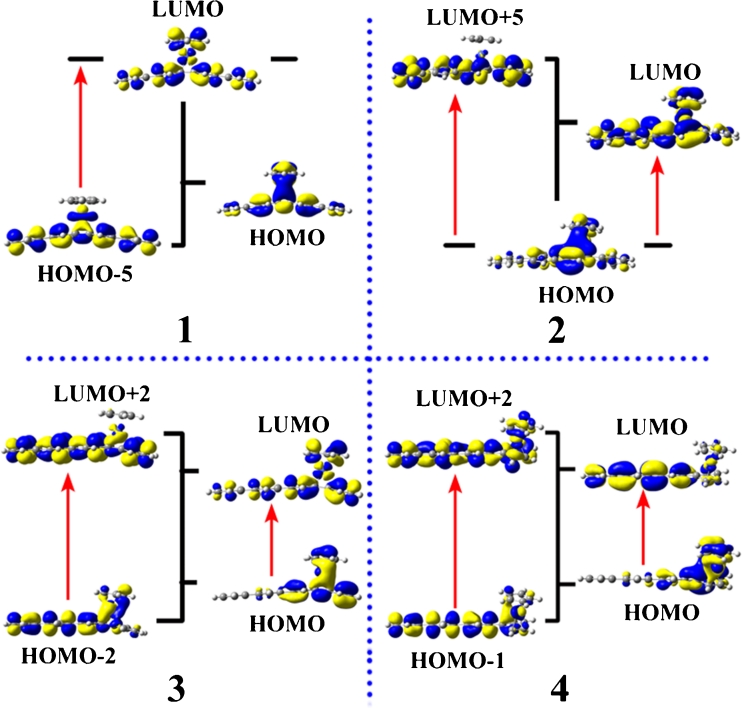
Major molecular orbital transitions of complexes 1–4

The first excited states of complexes 1–4 are generated by one-electron transfer from the highest occupied molecular orbital (HOMO) to the lowest unoccupied molecular orbital (LUMO). HOMO and LUMO are related to some chemical properties of complexes such as redox and electronic transition, etc. The HOMOs and LUMOs have been plotted in Fig. 4. It can be found that the HOMO of complex 1 is localized on the whole molecule, but the HOMOs of complexes 2–4 are localized around the Co and cyclopentadienyl. We take CpCo and linear [4]phenylene as two fragments to consider each fragment MO contributions (%) to the whole complex. The CpCo contribution to the HOMOs of complexes 1–4 are 18.48 %, 40.16 %, 29.50 %, 50.27 %, respectively. It suggests that CpCo acts as an important role in the CpCo linear [4]phenylene complexes. The LUMOs for complexes 1–3 are localized on the whole molecules. Whereas, the LUMO of complex 4 is localized on linear [4]phenylene and Co atom. However, oscillator strengths of the first excited states for the CpCo linear [4]phenylene complexes are small, which can be ignored. The crucial excited state with substantial oscillator strength is employed to observe the CT transitions clearly. As shown in Fig. 4, the major CT transition of complex 1 is from HOMO-5 to LUMO. The HOMO-5 of complex 1 is mainly localized on the linear [4]phenylene fragment and Co atom. The MO contribution of CpCo on HOMO-5 is 29.84 %, while the Co atom for MO contribution is 29.55 %. It implies that the significant CT transitions is mainly from Co atom to linear [4]phenylene and then to cyclopentadienyl. Specifically, these CT transitions are assigned to d (Co) → π^*^ (linear [4]phenylene) and π (linear [4]phenylene)→π^*^ (linear [4]phenylene) transitions. The major CT transition of complex 2 is from HOMO to LUMO+5. The LUMO+5 of complex 2 is almost localized on the linear [4]phenylene fragment (99.00 %). This CT is assigned to d (Co)→π^*^ (linear [4]phenylene). The major CT of complex 3 is from HOMO-2 to LUMO+2. The HOMO-2 and LUMO+2 of complex 3 are almost localized on all the linear [4]phenylene fragment, too (73.36 % and 96.11 %, respectively). The major CT transition of complex 4 is from HOMO-1 to LUMO+2. HOMO-1 and LUMO+2 of complex 4 are similar to HOMO-2 and LUMO+2 of complex 3, respectively. This illustrates that there are π→π^*^ transition in linear [4]phenylene and d→π^*^ transition between Co atom and linear [4]phenylene. And this is an important reason to produce second-order NLO response for all studied complexes.

### Second-order NLO property

**Fig. 5:**
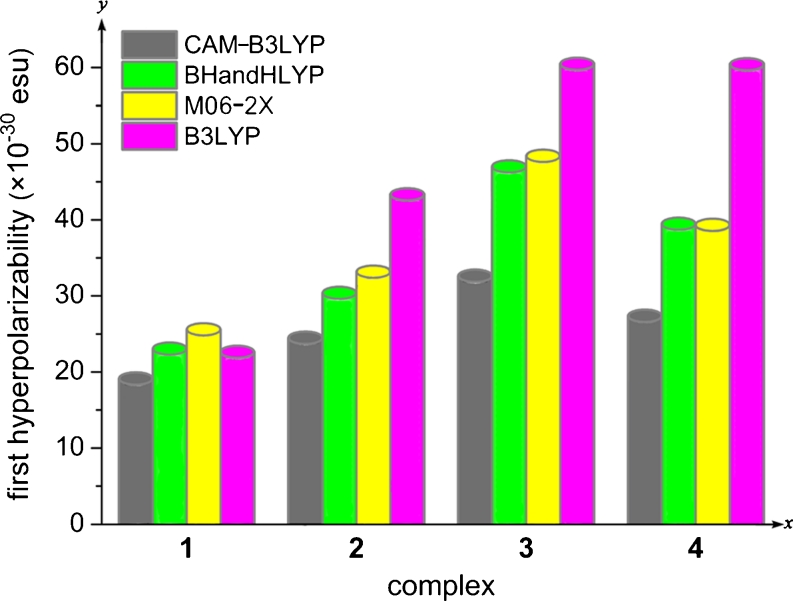
The tendency of *β*_*tot*_ values for complexes 1–4

The calculated *β*_*tot*_, *β*_*x*_, *β*_*y*_ and *β*_*z*_ values of complexes 1–4 at four different functionals have been listed in Table 4 and the variation tendency of *β*_*tot*_ values has been plotted in Fig. 5. From Table 4, among complexes 2–4, the *β*_*x*_ values dominate the second-order NLO response, because the skeleton atoms mostly locate on the *x*-axis, and the variation tendency of *β*_*x*_ values for the studied complexes is similar to their *β*_*tot*_ values. The *β*_*y*_ values also significantly contribute to the second-order NLO responses. This indicates that CT transfer also occurs along *y*-axis. The *β*_*x*_ value of complex 1 is close to zero. But why does the *β*_*x*_ value of complex 1 become close to naught suddenly? To understand it, the structure–property relationship of complex 1 is taken into account. As we know, linear [4]phenylene is a centrosymmetric molecule. When the CoCp is in the middle of linear [4]phenylene, complex 1 is symmetric structure, and it is therefore sufficient for revealing the relationship between the *β*_*x*_ value and the geometrical structure.

**Table 4:** The *β*_*tot*_, *β*_*x*_, *β*_*y*_ and *β*_*z*_values (×10^−30^ esu) for complexes 1–4 have been obtained by four methods. Effect of solvent on first-order hyperpolarizability has also been obtained by CAM-B3LYP method at the polarizable continuum model (PCM) in the presence of THF solution

Complex	Method	*β*_*x*_	*β*_*y*_	*β*_*z*_	*β*_*tot*_
1	CAM-B3LYP	−0.003	−19.152	0.104	19.152
	CAM-B3LYP (PCM)	−0.014	−48.413	0.291	48.414
	BHandHLYP	−0.005	−23.094	0.067	23.094
	M06-2X	−0.004	−25.601	0.111	25.602
	B3LYP	−0.002	−22.655	0.100	22.655
2	CAM-B3LYP	18.691	−13.953	−7.539	24.513
	CAM-B3LYP (PCM)	39.734	−35.344	−19.226	56.548
	BHandHLYP	23.502	−17.210	−8.689	30.398
	M06-2X	25.135	−19.160	−10.227	33.218
	B3LYP	39.634	−15.517	−8.535	43.410
3	CAM-B3LYP	29.218	−14.567	−0.749	32.656
	CAM-B3LYP (PCM)	59.950	−37.132	−1.583	70.535
	BHandHLYP	43.045	−18.970	−1.121	47.053
	M06-2X	43.664	−21.010	−1.159	48.470
	B3LYP	58.468	−15.624	−1.433	60.536
4	CAM-B3LYP	24.098	−11.533	6.134	27.411
	CAM-B3LYP (PCM)	44.407	−28.185	15.675	54.882
	BHandHLYP	34.964	−16.418	8.360	39.521
	M06-2X	33.757	−17.848	9.537	39.357
	B3LYP	57.789	−15.519	8.889	60.494

For CAM-B3LYP functional results, due to the change of CpCo location along the linear [4]phenylene, there is a stepwise escalation for the *β*_*tot*_ values of CpCo linear [4]phenylene complexes: 1<2<4<3. It illustrates that the second order NLO responses could be tuned by changing the location of CpCo. The solvent effect is also taken into account for the first hyperpolarizabilitiy. The static first hyperpolarizabilities are calculated in THF solvent by CAM-B3LYP functinonal (see Table 4). The results show that the hyperpolarizabilities are in the range of ~2.0 to ~2.5 times larger than that of calculations in vacuo. However, the order of hyperpolarizabilities for the studied complexes is still in accordance with the calculations in vacuo.

To have an insight into the origin of second-order NLO responses and explain the position effect of CpCo on NLO responses, we consider the widely used two-level model established by Oudar and Chemla [37]. The two-level model expression is defined as:

4 $$ {{{\beta \propto \varDelta {\mu_{gm }}{f_{os }}}} \left/ {{\varDelta E_{gm}^3}} \right.}, $$

Where ∆*μ*_*gm*_ is the difference between excited and ground state dipole moments, *f*_*os*_ is the oscillator strength, ∆*E*_*gm*_ is the transition energy. From the expression, the *β* value is inversely proportional to the cube of transition energy. Obviously, the transition energy is the decisive factor in the *β* value.

The crucial transition energies of the four complexes were obtained by the TDDFT at pure functional BP86. The crucial transition energies show a descending trend for complexes 1–4: 1(3.22 eV) > 2(3.13 eV) > 4(2.72 eV) > 3(2.70 eV), which is inversely proportional to the *β*_*tot*_ values of CpCo linear [4]phenylene complexes 1–4. In addition to this, the descending trend of crucial transition energies explains the gradually increase of *β*_*tot*_ values as the location of the CpCo changes. As a result, changing the position of CpCo can obtain a good NLO candidate.

### Frequency-dependent second-order NLO properties

The frequency-dependent NLO properties of the CpCo linear [4]phenylene complexes are evaluated by couple-perturbed (CP) DFT method with CAM-B3LYP functional, and the same basis sets with static *β*_*tot*_ value calculation are used. We calculated the frequency dependent first hyperpolarizability *β*(−2*ω*;*ω*,ω) for the second harmonic generation (SHG) and *β*(−*ω*;*ω*,0) for the electro-optical Pockels effect (EOPE). It is very important to choose an appropriate frequency *ω* to calculate frequency-dependent NLO properties of these CpCo linear [4]phenylene complexes. Generally, the molecular hyperpolarizabilities, *β*(−2*ω*;*ω*,ω), have been measured in a fundamental incident wavelength which has a second harmonic far enough from the absorption bands to avoid the overmeasure of *β* values due to resonance effects. Hence, two near resonant wavelength of *ω* = 0.0428 a.u. (1064 nm) and *ω* = 0.0340 a.u. (1340 nm), one nonresonant wavelength of *ω* = 0.0239 a.u. (1907 nm) are adopted to compute the frequency-dependence. As shown in Table 5, the magnitude of the frequency-dependent first hyperpolarizability increases with the increasing frequency. The values of *β*(−2*ω*;*ω*,ω) in the CpCo linear [4]phenylene complexes are larger than that of *β*(−*ω*;*ω*,0), and both of the *β*(−2*ω*;*ω*,ω) and *β*(−*ω*;*ω*,0) values are larger than the corresponding static *β*_*tot*_ values. Thus, *β*(−2*ω*;*ω*,ω) exhibits the largest frequency dispersion. Since the switching between structures from complex 1 to complex 3 is induced by light at *ω* = 0.1300 a.u. (350 nm), we also calculated the *β*(−2*ω*;*ω*,ω) values of complexes 1 and 3 at this wavelength. The *β*(−2*ω*;*ω*,ω) value of complex 3 is 8.8 times as large as that of complex 1 (see Table 5). The *β*(−2*ω*;*ω*,ω) values for complexes 1, 3 at *ω* = 0.1300 a.u. and for complexes 2, 4 at *ω* = 0.0428 a.u. are significantly enhanced, which could be caused by a resonant effect due to the weak absorption around second harmonic. Meanwhile, the frequency-dependent hyperpolarizabilities in THF were calculated using CPDFT method with CAM-B3LYP functional for complexes 1–4. As shown in Table 4, the solvent also leads to a slight enhancement of the frequency-dependent hyperpolarizability for each complex relevant to its frequency-dependent hyperpolarizability in vacuo.

**Table 5:** The frequency-dependent *β* values (×10^−30^ esu) and effect of solvent (in the THF solution) on frequency-dependent *β* values for complexes 1–4 have been evaluated by couple-perturbed (CP) DFT method, CAM-B3LYP functional

Complex	0.0239(a.u.)		0.0340(a.u.)		0.0428(a.u.)	
	*β*(−*ω*;*ω*,0)	*β*(−2*ω*;*ω*,ω)	*β*(−*ω*;*ω*,0)	*β*(−2*ω*;*ω*,ω)	*β*(−*ω*;*ω*,0)	*β*(−2*ω*;*ω*,ω)
1^a^	20.238	22.827	21.487	27.841	23.088	32.297
1(PCM)	37.664	35.799	40.197	44.306	43.472	52.602
2	26.664	35.904	29.129	54.323	31.934	411.106
2(PCM)	46.130	52.757	51.004	84.878	57.066	704.092
3^b^	35.088	40.805	37.838	73.428	41.320	46.439
3(PCM)	57.703	58.558	62.563	119.442	68.782	71.459
4	30.018	37.059	33.085	53.651	37.132	105.378
4(PCM)	47.106	51.893	52.408	77.772	59.560	164.025

a: The *β*(−2*ω*;*ω*,ω) value of complex 1 at *ω* = 0.1300 a.u. is 26716.925 × 10^−30^ esu. b: The *β*(−2*ω*;*ω*,ω) value of complex 3 at *ω* = 0.1300 a.u. is 234770.958 × 10^−30^ esu

### Switchable NLO response with larger β value contrast

In this section, reversibly switchable NLO behaviors were investigated in detail. To obtain NLO switches, apart from the reversible regulating measure, their *β* values contrast must be obvious. As discussed in “Geometrical structure and natural bond orbital (NBO) analysis” section, complexes 1 and 3 are more stable, which means that they are easier to be obtained and this explains why Vollhardt only got the CpCo linear [4]phenylene complexes 1 and 3 [24]. As listed in Table 4, the *β*_*tot*_ value of complex 1 is 19.152 × 10^−30^ esu calculated by CAM-B3LYP functional, while the value of complex 3 becomes larger (32.656 × 10^−30^ esu). The *β*_*tot*_ value of complex 3 is larger, so we consider complex 3 as the “ON” state. Conversely, we forecast complex 1 as the “OFF” state. Therefore, the CpCo linear [4]phenylene complexes 1 and 3 display NLO switching properties when the CpCo migrates along the linear [4]phenylene between the inner and outer cyclobutadiene ring, and we believe that CpCo linear [4]phenylene complexes are promising in switchable NLO materials.

## Conclusions

The geometrical structures and second-order NLO properties of CpCo linear [4]phenylene complexes were investigated by DFT methods. The theoretical investigation on geometrical structure suggests that the incorporation of CpCo gives rise to a significant distortion of planar linear [4]phenylene. Meanwhile, the NBO analysis demonstrates that the bond between Co and C atom is comprised of p orbital and d orbital, and the NBO charge demonstrates that CT occurs mainly from the Co atom to linear [4]phenylene. The electronic absorption spectra were investigated by the TDDFT methods. According to the TDDFT calculations, the d→π* and π→π* CTs can contribute to enhance the second-order NLO responses, and the order of the low transition energy for CpCo linear [4]phenylene complexes is inversely to their second-order NLO responses. Moreover, for the DFT results, due to the change of CpCo location along the linear [4]phenylene, there are large variations in the *β*_*tot*_ values of CpCo linear [4]phenylene complexes. The frequency-dependent first hyperpolarizabilities of the CpCo linear [4]phenylene complexes are also evaluated. From the results, *β*(−2*ω*;*ω*,ω) exhibits the largest frequency dispersion and the magnitude of the first hyperpolarizabilities increase with increasing frequency. In addition, the solvent leads to a slight enhancement of the hyperpolarizability and frequency-dependent hyperpolarizability.

These studies propose a novel field, wherein the *β*_*tot*_ value can be changed obviously by migrating the CpCo between the inner and the outer cyclobutadiene rings. We hope this new type of switchable NLO material based on CpCo linear [4]phenylene complexes will be developed rapidly in the future.
